# Neglected Microbes in Floral Nectar: Influence of Filamentous Fungi on Nectar Scent and Parasitoid Olfactory Responses

**DOI:** 10.1007/s10886-025-01586-2

**Published:** 2025-03-12

**Authors:** Jay Darryl L. Ermio, Ezio Peri, Salvatore Guarino, Patrizia Bella, Stefano Colazza, Bart Lievens, Michael Rostás, Antonino Cusumano

**Affiliations:** 1 Department of Agricultural, Food and Forest Sciences, University of Palermo, Palermo, 90128 Italy; 2 Department of Pest Management, Visayas State University, Baybay City, 6521 Philippines; 3 Institute of Biosciences and Bioresources (IBBR), National Research Council of Italy (CNR), Palermo, 90129 Italy; 4 Department of Microbial and Molecular Systems, KU Leuven, Leuven, B-3001 Belgium; 5 Department of Agricultural Entomology, University of Goettingen, 37077 Goettingen, Germany

**Keywords:** Floral Nectar fungi, Parasitoid foraging behavior, *Fagopyrum esculentum*, *Trissolcus basalis*, *Ooencyrtus telenomicida*

## Abstract

Floral nectar is a sugar-rich resource which is ubiquitously inhabited by a wide array of microorganisms. Fermentation by nectar-inhabiting microbes can alter several nectar traits, including nectar scent, via changes in the blend of volatile organic compounds (VOCs). Although there is growing evidence on how yeasts and bacteria influence the foraging behavior of flower-visiting insects, the potential role of other microbial taxa that can colonize nectar has been largely neglected. In this study, we investigated how filamentous fungi isolated from the floral nectar of buckwheat, *Fagopyrum esculentum*, affect nectar scent and the olfactory responses of two co-occurring egg parasitoid species, *Trissolcus basalis* and *Ooencyrtus telenomicida*. Among nectar-feeding insects, adult parasitoids are common visitors of flowers as they depend on sugar-rich resources to satisfy their energetic and nutritional needs. In olfactometer assays, we found that nectar fermentation by two out of six fungal strains, namely *Cladosporium* sp. SAAF 22.2.11 and *Cladosporium* sp. SAAF 22.3.29, elicited a behavioral response in the egg parasitoid species when tested against non-fermented nectar. In particular, *O. telenomicida* displayed positive olfactory responses to both *Cladosporium*-fermented nectars, while *T. basalis* only responded positively to nectar fermented by *Cladosporium* sp. SAAF 22.2.11. Chemical investigations revealed significant differences in the VOC blends across all six fungus-fermented nectars, despite a partial overlap between the blends emitted by attractive *versus* non-attractive nectars. Altogether, these findings highlight previously unexplored interactions between flower-visiting insects and flower-associated microbes, broadening our understanding beyond the well-studied influences of yeasts and bacteria.

**Supplementary Information:**

The online version contains supplementary material available at 10.1007/s10886-025-01586-2.

## Introduction

Floral nectar is a sugar-rich aqueous substance that stems from the phloem and is secreted by most flowering plants across the angiosperm communities (De la Barrera and Nobel 2004; Parachnowitsch et al. 2019). It has been recognized as an essential factor in mediating mutualistic interactions between flowering plants and nectar-feeding insects (Parachnowitsch et al. 2019; Schaeffer et al. 2017). Nectar is rich in carbohydrates, but also contains amino acids, minerals and vitamins essential for the insects’ metabolic activities (Kowalska et al. 2022; Lievens et al. 2015; Sobhy et al. 2018; Winkler et al. 2009). Many insects take advantage of the floral nectar, including pollinators such as honeybees and bumblebees, as well as natural enemies of insect pests like parasitoids (Géneau et al. 2012; Jervis et al. 1993; Vannette 2020). Conventionally, in intensive agricultural landscapes with limited nectar resources, non-crop habitats of nectar-rich flowering plants are established to enhance the effectiveness of natural enemies as biological control agents (Araj et al. 2011; Foti et al. 2017; Nafziger and Fadamiro 2011; Takasu and Lewis 1995).

It is well known that flowering plants release plumes of volatiles to attract flower visitors (Colazza et al. 2023; Raguso 2008). These floral volatiles are considered to be signals that help flower visitors locate and feed on floral nectar (Bianchi and Wäckers 2008; Foti et al. 2017; Raguso 2008; Schiestl 2015). However, in recent decades, it has become increasingly clear that floral nectar is ubiquitously inhabited by an array of microbes from different guilds, which may reshape the flowers’ distinctive odor signature as a product of microbial metabolism and, in turn, influence the foraging behavior of flower visitors (Klaps et al. 2020; Lievens et al. 2015; Pozo et al. 2014; Steffan et al. 2024; Vannette 2020). It has been suggested that nectar-inhabiting microbes have a dual effect on the composition of volatile organic compounds (VOCs) associated with floral nectar: first, they can modify the constitutive blends of VOCs emitted by sterile floral nectar; second, their metabolic activity can lead to the emission of *de novo* volatiles in nectar, also known as ‘microbial volatile organic compounds’ (mVOCs) (Cusumano and Lievens 2023). Aside from modifying nectar odors, nectar microbes may also be responsible for several alterations in other nectar traits, such as variations in the amount and composition of different sugars and amino acids, changes in pH, reduction of secondary metabolites, and even the formation of warmer and less viscous nectar (De Vega and Herrera 2013; Herrera et al. 2008; Herrera and Pozo 2010; Vannette et al. 2013; Vannette and Fukami 2016, 2018). Considering all these microbe-mediated changes in nectar traits, it is important to consider nectar-inhabiting microbes as “hidden players” that can affect the complex interactions between flowering plants and their associated insect visitors.

Bacteria and yeasts in nectar are the most widely studied microorganisms known to influence the foraging behavior of pollinators and parasitoids through changes in the nectar scent (Cusumano et al. 2023; Herrera et al. 2009; Lievens et al. 2015; Sobhy et al. 2018). For instance, the nectar specialist yeasts belonging to the genus *Metschnikowia* spp. are often reported to elicit positive responses in pollinators, especially in bumblebees (Herrera et al. 2013; Schaeffer et al. 2014, 2017). On the contrary, bacterial colonization of floral nectar can reduce, enhance or have a neutral effect on nectarivore behavior (Good et al. 2014; Junker et al. 2014; Rering et al. 2018). In the case of parasitoids, volatiles from the nectar specialist yeasts *Metschnikowia* spp. were reported to be attractive to the aphid parasitoid *Aphidius ervi* (Sobhy et al. 2018) and the egg parasitoids *Trissolcus basalis* and *Ooencyrtus telenomicida* (Ermio et al. 2024). More recently, *T. basalis* has also been reported to respond positively to nectar-inhabiting bacteria (Cusumano et al. 2023).

While an increasing body of evidence is accumulating on how yeasts and bacteria affect pollinators’ and parasitoids’ foraging behavior, the role of other microbial taxa that can potentially colonize nectar has been largely overlooked so far. Filamentous fungi of the genera *Cladosporium* and *Aspergillum* have also been reported to colonize sugar-rich environments such as floral nectar (Ehlers and Olesen 1997; von Arx et al. 2019), yet the role played by filamentous fungi in the interactions between flowering plants and insect visitors has been generally neglected. Given that filamentous fungi are closely related to yeasts, it is reasonable to hypothesize that filamentous fungi may also alter the properties of floral nectar through metabolic fermentation, with potential consequences for the community of flower-visiting insects. However, since filamentous fungi possess distinctive traits compared to yeasts, it can be expected that their effects on nectar traits and the foraging behavior of flower-visiting insects may differ from those induced by yeasts. Furthermore, several filamentous fungi have also been shown to be pathogens of hymenopterans (Nicoletti et al. 2024) and in some cases they may produce VOCs that mimic flowers to attract dispersal agents (Raguso and Roy 1998).

To further explore the ecological role of nectar-associated microbes beyond yeasts and bacteria, we first isolated and characterized the culturable filamentous fungi inhabiting the floral nectar of buckwheat (*Fagopyrum esculentum*), a flowering plant widely used in agricultural landscapes to enhance the effectiveness of parasitoids as natural enemies of insect pests (Gurr et al. 2017). Surprisingly, despite the importance of buckwheat in conservation biological control as a food source for many beneficial insects, its nectar-associated microbial community has not been investigated in detail, with the exception of bacteria (Cusumano et al. 2023). Subsequently, we investigated how fermentation by these filamentous fungi affects nectar scent and, in turn, the olfactory responses of two egg parasitoids, *T. basalis* and *O. telenomicida*. Both parasitoids are well known to feed on floral nectar (Foti et al. 2017, 2019), making them suitable organisms for investigating how microbes that colonize nectar influence insect foraging behaviors. The egg parasitoid *T. basalis* is a key natural enemy of the southern green stink bug *Nezara viridula*, a highly destructive insect pest of various vegetable crops worldwide (Esquivel et al. 2018). The egg parasitoid *O. telenomicida* is also associated with the stink bug *N. viridula*, and it has been reported to coexist with *T. basalis* in Southern Italy (Peri et al. 2014). Finally, to explore potential compounds involved in the parasitoids’ olfactory responses, we analyzed the chemical composition of the VOCs emitted by the fungus-fermented nectars.

## Methods and Materials

*Plant Rearing and Floral Nectar Sampling*. Buckwheat seeds were sown in 10-cell plug trays filled with commercial potting mix (Supernutrient Vegetable Soil, Virgoplant, Piacenza, Italy). Trays were placed in a climate-controlled chamber (24 ± 2 °C, 45 ± 10% RH, 12L:12D photoperiod). After germination, 1-week-old seedlings were transplanted to plastic pots (1-liter volume) and transferred to the experimental fields of the University of Palermo (Italy) where they were exposed - from May to October 2021 - to the natural community of insects and microbes in the area. This approach, which was repeated every month by sowing new seeds, aimed to capture the temporal diversity of microbes sampled from floral nectar, as microbial colonization may change over time. The first week of each month, floral nectar was sampled from plants at full bloom that were approximately 4–5 weeks old. On the day of collections, plants were transported to the laboratory early in the morning where nectar was collected from fully opened flowers. Nectar was sampled under sterile conditions in a laminar flow cabinet with the aid of 0.5 µL glass capillary tubes (see Cawoy et al. 2008 for a detailed description of the sampling procedure) and transferred to 1.5 mL Eppendorf tubes previously filled with 100 µL of sterile water. In each tube, nectar from 50 flowers (10 flowers/plant) was collected (~ 0.05 µL of nectar/flower), and a total of 20 samples for each timepoint were prepared for isolation of nectar fungi.

*Isolation and Identification of Filamentous Fungi from Buckwheat Nectar*. Diluted flower nectar samples (100 µL) were spread evenly onto a Petri plate with yeast extract glucose agar (YGC, Sigma-Aldrich, Steinheim, Germany) supplemented with 0.5 g/L chloramphenicol (BioChemica, ITW Reagents SRL, Milan), to determine the presence of filamentous fungi. Then, agar plates were incubated at 25 °C until fungal growth was observed. Afterwards, morphologically distinct fungal colonies from the different plates were subcultured in separate plates using the same medium. Monosporic cultures of the fungal isolates were stored in test tube slants with potato dextrose agar (PDA) at 4 °C until further use.

All isolates were identified morphologically and further confirmed through molecular analysis of the internal transcribed spacer regions of the fungal ribosomal DNA (ITS rDNA). For fungal DNA extraction, mycelia and conidia were harvested by gently scraping fungal material from 7-15-days old cultures grown on PDA plates with the aid of a disposable sterile plastic wire loop. Harvested fungal material was then placed in 2-ml Eppendorf tubes along with glass beads and vortexed to disrupt fungal cells. Then, 600 ml CTAB extraction buffer (pH 8-8.4) was added to the tubes and vortexed for 5–10 min. Next, the suspension was incubated in a thermal block at 65 °C for 1 h and mixed by vortexing every 10–15 min. After incubation, 300 µl of phenol and 300 µl of chloroform: isoamyl alcohol (24:1) solution were added to each tube, which was then gently inverted several times to mix. All tubes were centrifuged at 9,000 x g for 10 min. The supernatant was then transferred to a 1.5-ml Eppendorf tube containing 600 µl chloroform: isoamyl alcohol (24:1) solution and briefly mixed and centrifuged at 9,000 x g for 10 min. Following centrifugation, the supernatant was transferred to a new 1.5-ml Eppendorf tube, and 600 µl of isopropanol was added, followed by brief shaking. The tubes were then incubated at -20 °C for 10–20 min and centrifuged again at 17,000 x g for 10 min. The supernatant was discarded, and the remaining DNA pellets were washed twice with 70% alcohol. The tubes were placed in a laminar flow cabinet to allow the DNA pellets to air dry, then resuspended in 100 µl of sterile distilled water. Finally, DNA concentrations were measured using a NanoDrop^®^ ND-1000 spectrophotometer, and samples were stored at -20 °C for later analysis.

Fungal ITS rDNA was amplified using the primer pair ITS1F (CTTGGTCATTTAGAGGAAGTAA) (Gardes and Bruns 1993) and ITS4 (5’-TCCTCC GCTTATTGATATGC-3’) (White et al. 1990). Polymerase chain reaction (PCR) was run in a 25-µl volume PCR mixture with 1× of the GoTaq^®^ G2 colorless master mix, 2 µM of the forward primer ITS1F, 2 µM of the reverse primer ITS4, 1.5 µl of water and 50 ng of fungal DNA template. The reaction was carried out using the MultiGene OptimaxTM Gradient thermal cycler (Labnet International, Inc.) programmed with an initial denaturation at 94 °C for 3 min, 35 cycles of denaturation-annealing-extension reactions at 95-55-72 °C for 30-30–45 s, and the last cycle at 72 °C for 10 min for the final extension. Gel electrophoresis (1.0% agarose gel) was performed to check correct PCR amplification of the target fragment. Samples were then sent to BMR Genomics (Padova, Italy, https://www.bmr-genomics.it/) for DNA sequencing using the reverse primer ITS4. Finally, fungal DNA sequences were edited and compared with reference sequences of type strains in the GenBank standard database with the help of BLASTN (Basic Local Alignment Search Tool, https://www.ncbi.nlm.nih.gov/). Fungal isolates were assigned to the closest homologous strain(s) based on the highest percentage of sequence identity. Additionally, sequences obtained in this study along with 18 reference sequences retrieved from GenBank were aligned using Clustal W in MEGA XI software and phylogenetic analysis inferred with the Neighbor-joining (NJ) method based on the Jukes-Cantor model. All sequences obtained in this study have been deposited in GenBank under the accession numbers PP990345- PP990350 (Table S1).

To confirm if the isolated filamentous fungal strains are able to grow and thrive in floral nectar, we conducted a growth trial using synthetic nectar solutions with different concentrations of sucrose (15%, 30%, and 50%) supplemented with casamino acids in accordance with the protocol outlined below. Fungal growth was checked visually after 5 days of fermentation. Additionally, 100 µl of the fermentation medium was plated on PDA to confirm the viability of the fungi. All fungal strains tested were proven to be growing well in the different nectars regardless of the sucrose concentrations.

*Synthetic Nectar Solutions*. To assess the impact of the fungal strains isolated from buckwheat nectar on parasitoid olfactory responses, synthetic nectar solutions were prepared by mixing filter-sterilized 50% (w/v) sucrose (Carlo Erba Reagents S.A.S., Val-de-Reuil, France) solution and 3.16 mM casamino acids (OmniPur, Merck KGaA, Darmstadt, Germany) as outlined by Vanette and Fukami (2014). This solution mimics several floral nectars found in nature, including buckwheat nectar (Cawoy et al. 2006). To produce fungus-fermented synthetic nectars, synthetic nectar solutions were individually inoculated with the six fungal strains. In brief, mother cultures were plated on PDA and incubated for 7 days at 25 °C. Then, fungal conidia were harvested in sterile distilled water with the aid of a disposable sterile plastic wire loop. The resulting suspension was subsequently filtered using sterile cloth gauze to remove traces of mycelium. The number of conidia was counted using the Thoma hemocytometer to ensure the consistency of the conidial counts across the different fungal suspensions for nectar inoculation. A fresh 100 µL aliquot of 10⁶ conidia/ml fungal suspension for each fungal strain, including the negative control, was inoculated into 20 ml of sterile synthetic nectar solution. The tubes were then incubated at 25 °C for 5 days, reflecting the approximate floral lifespan of many plant species (Lenaerts et al. 2016, 2017; Peay et al. 2012; Vannette and Fukami 2014). After the incubation period, the fungus-fermented synthetic nectars were centrifuged at 10.000 x g, and the supernatants were filtered through 0.20-micron filters to obtain sterile fungus-fermented media. To confirm the absence of fungal contamination, filtered fungus-fermented synthetic nectars were streaked onto PDA plates, and fungal growth was monitored over 5 days. Finally, the sterile fungus-fermented synthetic nectars were aliquoted into 1-ml amber vials and stored at -80 °C for further use and subsequent volatile analyses. All fermentations were performed in five biological replicates which were carried out across different days using a randomized experimental design.

*Parasitoid Rearing*. The egg parasitoids *Trissolcus basalis* (Hymenoptera: Scelionidae) and *Ooencyrtus telenomicida* (Hymenoptera: Encyrtidae) were maintained using fresh *Nezara viridula* egg masses obtained as described below. Diluted organic honey (80:20 v/v) was provided to the adult wasps. The *N. viridula* colony was maintained in the laboratory by feeding the insects with fresh organic vegetables (carrots, cabbages, tomatoes and green beans) and sunflower/soybean seeds. Nymphs and adults of the stinkbugs were reared separately in insect cages (47.5 × 47.5 × 47.5 cm, BugDorm-44545 MegaView Science Co. Ltd, Taichung, Taiwan) under controlled conditions of 24 ± 1 °C, 70 ± 5% RH and 1L:10D photoperiod. To obtain egg masses of the stinkbugs, tissue papers were either hung or scattered inside the net cages as an oviposition substrate. Egg masses were collected three times a week and were used to maintain both the stinkbug and egg parasitoid colonies. All insect colonies were originally established from specimens collected around Palermo, Italy (38°03’57"N, 13°28’10"E).

*Nezara viridula* egg masses were exposed to 2–3 gravid females of each parasitoid species for two days inside 55 ml glass tubes (25 × 150 mm). Subsequently, each parasitized egg mass was transferred to another 55 ml glass tube until all parasitoid emerged. After emergence, male and female wasps were kept together to allow mating. About 24 h before the experiments, newly emerged female wasps were individually placed in small vials (1.5 × 5 cm) without food to induce starvation.

*Olfactory Response of Egg Parasitoids to Fungus-Fermented Synthetic Nectar*. A four-chamber static olfactometer was used to investigate the fungus-mediated effects on the olfactory behavior of both stinkbug egg parasitoids when exposed to odors of fungus-fermented synthetic nectars. The olfactometer consisted of a cylindrical body made of acrylic glass (4.5 × 20 cm) with four equal divisions supported by vertical plates to isolate the odor sources, following the design described by Steidle and Schoeller (1997). To separate the wasps from the odor sources, a walking arena (1.5 × 20 cm) made of plastic gauze (0.5 mm mesh) fitted with an acrylic rim was placed on the top of the chambers. The olfactometer was also covered with a netted lid (~ 0.5 mm mesh) to confine the wasps inside the walking arena while also facilitating the vertical diffusion of odors to avoid possible volatile saturation in the chamber. Throughout the experiment, the olfactometer was placed on a rack covered with a blackout curtain equipped with a white fluorescent light (Philips, TLD 58 W/640) on the top. In each bioassay, fungus-fermented nectar was tested against control (non-fermented) nectar, by positioning both odor sources diagonally opposite each other in the olfactometer. Previous results have shown that *T. basalis* is attracted by odors from the freshly collected nectar of buckwheat over non-fermented synthetic nectar (Cusumano et al. 2023). In each bioassay, 200 µl of either fermented or non-fermented nectar was pipetted onto sterile filter paper (3.8 cm diameter, Whatman No. 1) placed on a Petri dish within the odor chambers. One egg parasitoid was released inside the walking arena at a time and given at least 1 min to get accustomed to the arena. Subsequently, the behavior of the egg parasitoid was assessed and recorded for 5 min with the aid of a video camera (Logitech C920 HD Pro Webcam). The residence time spent by the egg parasitoid above the chambers was then analyzed using the event-recording software J Watcher V0.9 (https://www.jwatcher.ucla.edu/).

For each pairwise comparison, 40 egg parasitoids were tested, using a fully randomized experimental design. As the VOC composition of all five biological replicates of synthetic nectar was highly similar (see results), the olfactory response was determined for one of the five biological replicates. To avoid positional biases in the set-up, the odor sources and the olfactometer were rotated 90° after testing each insect. Also, the filter paper disks with the synthetic nectar were changed every replication. At the end of the day, the olfactometer was cleaned using 90% ethanol and tap water, followed by drying overnight at room temperature. All olfaction bioassays were carried out in a room with temperature and relative humidity kept at 21 ± 5 °C and 50 ± 10%, respectively, between 09h00 and 17h00, and each insect was only tested once. Every day about 20 observations were made.

*Volatile Analysis of Fungus-Fermented Synthetic Nectar*. The VOCs from the fungus-fermented synthetic nectars (*n* = 5) were sampled using the headspace solid-phase microextraction (HS- SPME) technique and analyzed by gas chromatography-mass spectrometry (GC-MS) using an Agilent 6890 GC system equipped with a DB5-MS column and a MS5973 quadruple MS. The system was programmed to operate in a splitless injection mode with a continuous flow of helium as carrier gas. The inlet port of the GC was set to a temperature of 260 °C, while the detector temperature was maintained at 280 °C. The initial temperature in the GC oven was set at 40 °C and held for 5 min, followed by a gradual increase of 10 °C/min until reaching 250 °C, where it was maintained for 30 min. Mass spectra were recorded over the range of 40 and 550 atomic mass units using an electron impact ionization energy of 70 eV.

The SPME fiber used for the experiment was Carbowax–divinylbenzene (CW-DVB), 65 μm (Supelco, Bellefonte, PA, USA). Before use, the fiber was conditioned in the GC inlet port for 30 min with a temperature of 200 °C. Samples were prepared by pipetting 100 µl of either fungus-free fermented synthetic nectar or non-fermented synthetic nectar onto a filter paper disk Whatman No. 1 (3.8 cm diameter) placed inside a 40-ml amber vial. The vial was sealed with a polytetrafluoroethylene-lined silicon septum and a screw cap. VOCs were then extracted from the headspace of the glass vial by inserting the SPME needle through the septum and exposing the fiber for 1 h after a stabilization period of 10 min. The fiber was then thermally desorbed in the GC inlet for 1 min, and analyses were performed using the set parameters described above.

MSD ChemStation was utilized to quantify the peak areas of the chromatogram while spectral libraries such as NIST 2011 and Wiley 17 and the online database Pherobase (www.pherobase.com) were utilized for compound identification. When available, commercial standards from Sigma-Aldrich (Milan, Italy) were injected for compound detection. Retention indices of the identified compounds were calculated based on the linear alkane standards (C7-C30) that were injected immediately prior to the experiment. Contaminants were excluded from the analysis by subtracting the background signals arising from the blank headspace of the empty vials used for volatile extraction (Eisen et al. 2022). Furthermore, given the non-volatile nature of the synthetic nectar recipe - which contains only water, sucrose, and amino acids - we considered volatiles emitted from the sterile nectar to be contaminants as well.

*Statistical Analysis*. Olfactometer data were analyzed with the R software, version R 4.4.0 (R Core Team 2024). Since residence time data were not normally distributed (Shapiro–Wilk normality test) the non-parametric Wilcoxon signed rank exact test was used. To analyze the peak areas of the VOCs, multivariate data analysis (Principal Component Analysis—PCA) was carried out using the software MetaboAnalyst (Xia et al. 2009). Data were log-transformed, mean-centered and subsequently scaled to unit variance before the analyses. Results were visualized with biplots combining together score plots (which reveal the sample structure according to model components) and loading plots (which display the contribution of the variable to these components).

## Results

*Isolation and Identification of Filamentous Fungi from Buckwheat Nectar*. In total, six morphotypes were retrieved from the buckwheat nectar samples. Molecular analysis of the ITS regions of the fungal ribosomal DNA indicated that the strains belonged to three families (Stachybotryaceae, Cladosporiaceae, and Aspergillaceae) of the phylum Ascomycota (Table S1). In the family of Stachybotryaceae, we found one strain of *Stachybotrys* sp. (SAAF 22.1.15), while three strains of Cladosporiaceae were found, belonging to the genus *Cladosporium* (SAAF 22.2.11, SAAF 22.2.12, and SAAF 22.3.29). Lastly, two *Aspergillus* strains (SAAF 22.3.26 and SAAF 22.4.40) belonging to the family Aspergillaceae were identified (Supplementary Table 1). Phylogenetic placement of the obtained sequences (550 bp) along with 18 reference sequences retrieved from the GenBank database confirmed their identification (Fig. S1). Yeasts were not detected in any of the samples analyzed, according to our morphological and molecular analysis.

### Olfactory Response of Egg Parasitoids Toward Fungus-Fermented Synthetic Nectar

*Trissolcus basalis*. Adult females of *T. basalis* significantly preferred the odors emitted by nectar fermented by *Cladosporium* sp. SAAF 22.2.11 over the non-fermented nectar (*Z* = 2.218, *df* = 39, *P* = 0.026). In contrast, no preferences were observed when wasps were exposed simultaneously to the odors of the non-fermented synthetic nectar and those fermented by *Cladosporium* sp. SAAF 22.2.12 (*Z* = 0.659, *df* = 39, *P* = 0.518), *Cladosporium* sp. SAAF 22.3.29 (*Z* = 0.820, *df* = 39, *P* = 0.420), *Aspergillus* sp. SAAF 22.3.26 (*Z* = 0.551, *df* = 39, *P* = 0.590), *Aspergillus* sp. SAAF 22.4.40 (*Z* = 1.102, *df* = 39, *P* = 0.276) and *Stachybotrys* sp. SAAF 22.1.15 (*Z* = 0.054, *df* = 39, *P* = 0.962)Fig. . 1a).

**Fig. 1:**
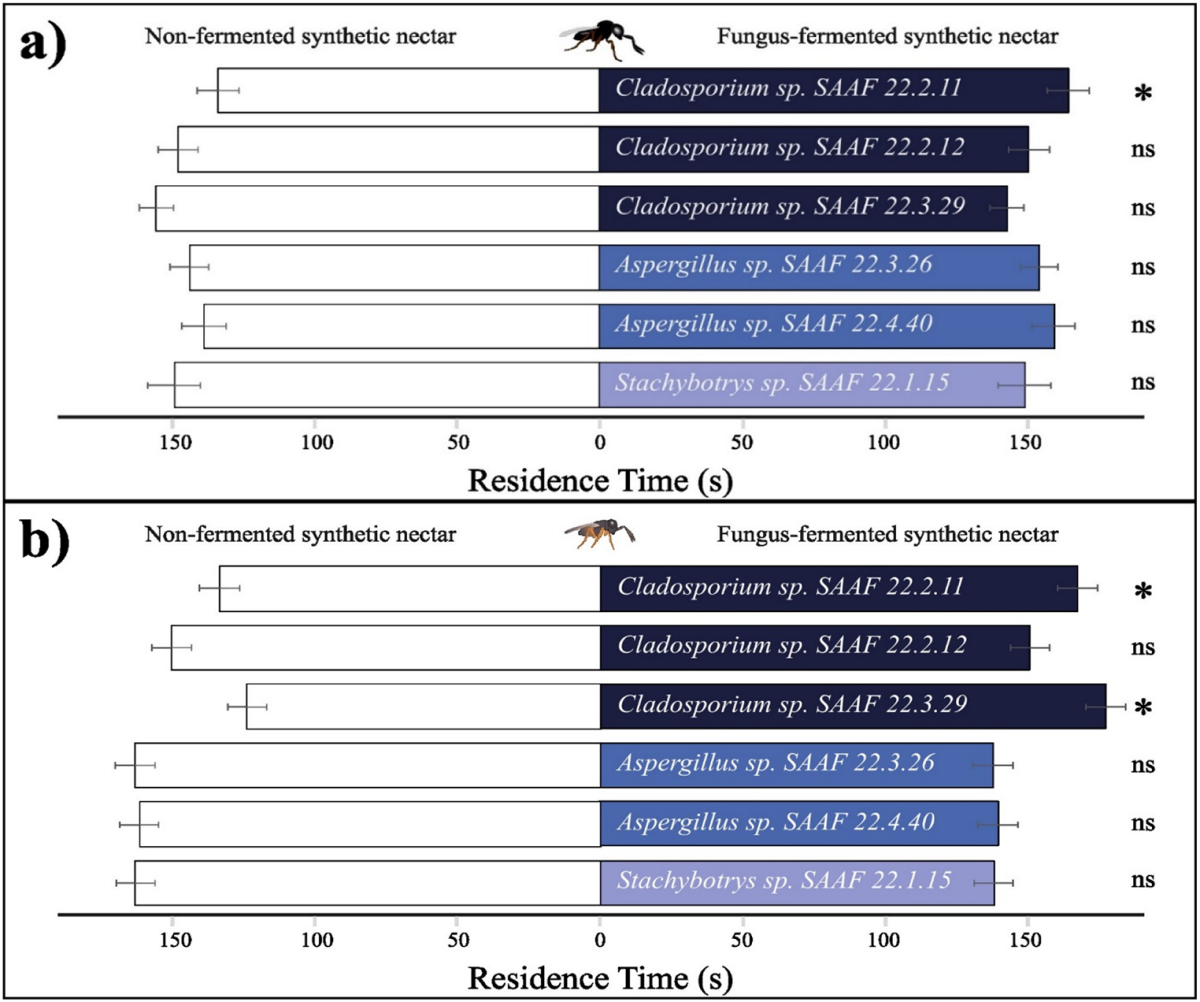
Olfactory responses of the adult female egg parasitoids **(a)***Trissolcus basalis* and **(b)***Ooencyrtus telenomicida* evaluated in terms of residence time (s) in two-choice bioassays over an observation time of 300 s. Test treatments represent synthetic nectars fermented by the filamentous fungi *Cladosporium* sp. SAAF 22.2.11, *Cladosporium* sp. SAAF 22.2.12, *Cladosporium* sp. SAAF 22.3.29, *Aspergillus* sp. SAAF 22.3.26, *Aspergillus* sp. SAAF 22.4.40 and *Stachybotrys* sp. SAAF 22.1.15 (all blue-shaded bars). Non-fermented synthetic nectar was used in all the pairwise comparisons as a control (white bars). Each treatment was replicated 40 times (nonparametric, Wilcoxon signed rank exact test, * = *P* < 0.05; ns = not significant). Error bars represent SE (standard error)

*Ooencyrtus telenomicida*. Adult females of *O. telenomicida* significantly preferred the odors emitted by the nectar fermented by *Cladosporium* sp. SAAF 22.2.11 (*Z* = 2.191, *df* = 39, *P* = 0.027) and *Cladosporium* sp. SAAF 22.3.29 (*Z* = 2.137, *df* = 39, *P* = 0.032) when compared to the non-fermented control nectar. No preferences were observed when *O. telenomicida* females were exposed simultaneously to the odors from the non-fermented nectar and those fermented by the other filamentous fungi, including *Cladosporium* sp. SAAF 22.2.12 (*Z* = 0.013, *df* = 39, *P* = 0.994), *Aspergillus* sp. SAAF 22.3.26 (*Z* = 1.095, *df* = 39, *P* = 0.276), *Aspergillus* sp. SAAF 22.4.40 (*Z* = 1.048, *df* = 39, *P* = 0.301) and *Stachybotrys* sp. SAAF 22.1.15 (*Z* = 1.035, *df* = 39, *P* = 0.307)Fig. . 1b).

*Volatile Analysis of Fungus-Fermented Synthetic Nectar*. Headspace analysis of the nectar fermented by the six filamentous fungi revealed a total of 7 VOCs. Of these, α terpineol, 2,4 dimethylglutarate, 2,6-di-tert-butyl-p-benzoquinone and heptadecane were present in all the strains tested. Differently, methyl benzoate was present in all the fungi except in *Stachybotrys* sp. SAAF 22.1.15 while hydroxyacetophenone was detected only in *Aspergillus* sp. SAAF 22.3.26 and in *Cladosporium* sp. SAAF 22.2.11 (for details, see Table 1). Based on the PCA, a significant separation of the volatile blends was found across all fungus-fermented synthetic nectars (permutation test *P* < 0.001) despite a partial overlap between the blends emitted by attractive and non-attractive nectarsFig. . 2). The first principal component explained 34.18% of the variation whereas the second component explained 26.6% of the variation.

**Table 1:** Volatile organic compound (VOC) composition of synthetic nectars fermented by the filamentous fungi *Aspergillus* Sp. SAAF 22.3.26, *Aspergillus* Sp. SAAF 22.4.40, *Cladosporium* Sp. SAAF 22.2.11, *Cladosporium* Sp. SAAF 22.2.12, *Cladosporium* Sp. SAAF 22.3.29, *Stachybotrys* Sp. SAAF 22.1.15

ID	Volatile Organic Compounds	RT (min)	RI	Synthetic nectar^a^					
				*Aspergillus *sp. SAAF 22.3.26	*Aspergillus* sp. SAAF 22.4.40	*Cladosporium *SAAF 22.2.11	*Cladosporium* SAAF 22.2.12	*Cladosporium* SAAF 22.3.29	*Stachybotrys *SAAF 22.1.15
1	Methyl benzoate	16.56	1090	3.26 ± 0.75	2.28 ± 1.36	2.85 ± 0.91	0.89 ± 0.27	1.05 ± 0.48	ND
2	Hydroxyacetophenone	18.65	1157	2.52 ± 1.18	ND	3.61 ± 0.82	ND	ND	ND
3	α Terpineol	19.79	1194	2.35 ± 0.40	2.54 ± 0.13	2.61 ± 0.68	2.26 ± 0.30	2.96 ± 0.36	3.19 ± 0.58
4	2,4-Dimethylglutarate	19.94	1199	3.21 ± 0.56	2.50 ± 0.72	2.97 ± 0.94	3.02 ± 0.49	2.35 ± 0.64	3.93 ± 1.12
5	2,6-Di-tert-butyl-p-benzoquinone	27.06	1461	5.31 ± 0.75	6.85 ± 0.82	4.65 ± 0.61	4.74 ± 1.42	6.47 ± 0.75	6.23 ± 1.75
6	Heptadecane	32.71	1700	2.09 ± 0.92	2.63 ± 1.12	3.77 ± 0.98	3.19 ± 1.53	6.43 ± 0.71	1.82 ± 0.98
7	Hexadecanoic acid	38.03	1957	7.10 ± 3.43	3.27 ± 2.41	ND	ND	ND	1.82 ± 0.85

^a^Values in the table are means of absolute peak areas (× 10^5^) ± SE (standard error) of five biological replicates (*n* = 5) of synthetic nectars fermented by six filamentous fungi (Aspergillus sp. SAAF 22.3.26, Aspergillus sp. SAAF 22.4.40, Cladosporium sp. SAAF 22.2.11, Cladosporium sp. SAAF 22.2.12, Cladosporium sp. SAAF 22.3.29, Stachybotrys sp. SAAF 22.1.15). Volatile organic compound(s) are ordered by their increasing retention time (RT) and retention index (RI). VOCs were tentatively identified based on spectra, Kovats retention index, NIST 2011, Wiley 17, and the “The Pherobase” library matches. ND: not detectedyb

**Fig. 2:**
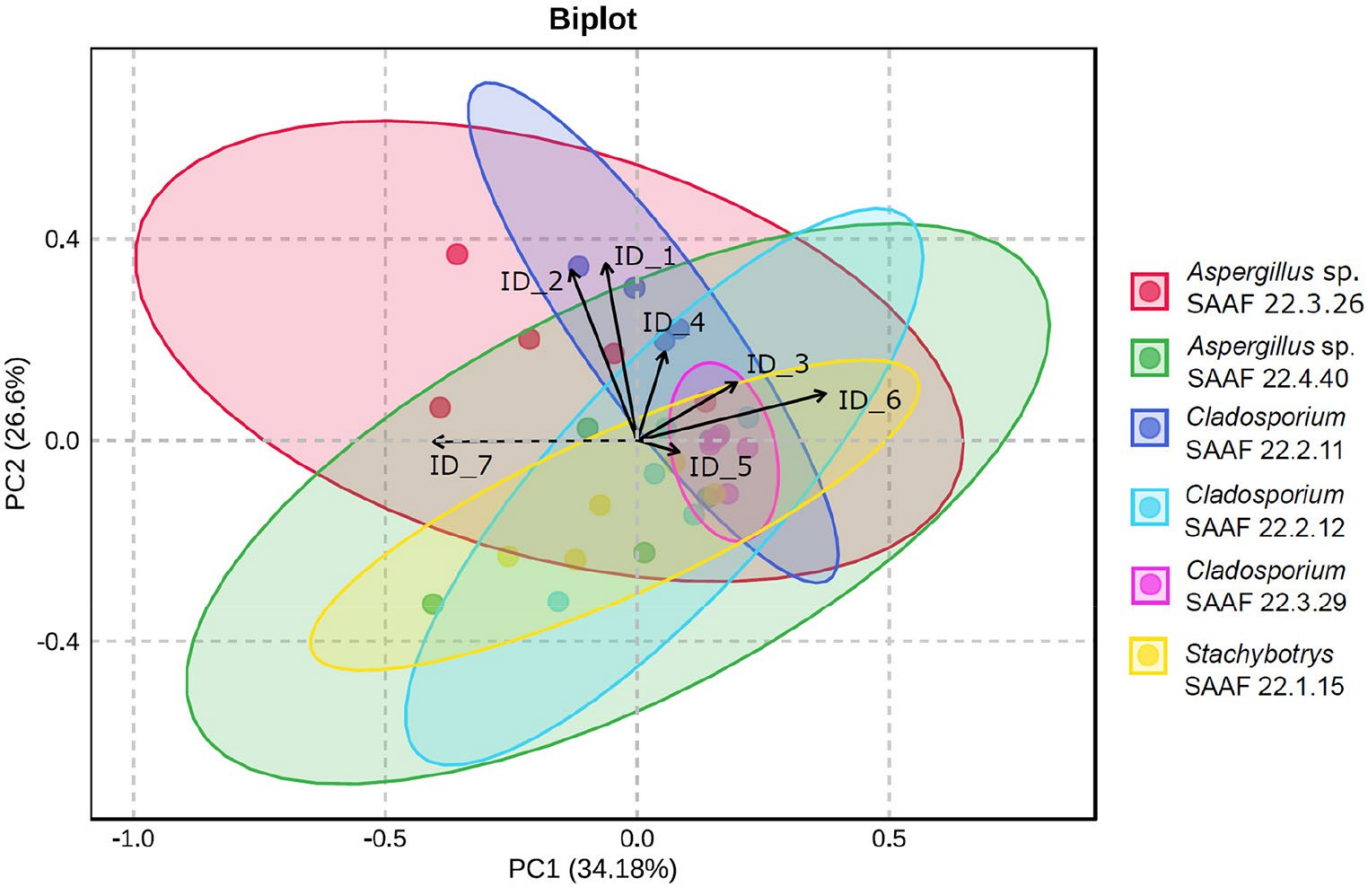
Principal Component Analyses (PCA) of synthetic nectar (*n* = 5) fermented by the filamentous fungi *Aspergillus* sp. SAAF 22.3.26, *Aspergillus* sp. SAAF 22.4.40, *Cladosporium* sp. SAAF 22.2.11, *Cladosporium* sp. SAAF 22.2.12, *Cladosporium* sp. SAAF 22.3.29, *Stachybotrys* sp. SAAF 22.1.15. In the byplot, the score values visualize the grouping pattern of the samples according to the first two principal components (PCs) with the explained variance in parenthesis. Ellipses indicate a 95% confidence interval. Arrows indicate the loading values of the first two PCs showing the contribution of each compound. See Table 1 for the list of compound IDs

## Discussion

In this study, we found that insect parasitoids can respond to changes in nectar odors caused by the fermentation of nectar-inhabiting filamentous fungi. From the floral nectar of buckwheat, we were able to culture six different strains of filamentous fungi belonging to three families (Stachybotryaceae, Cladosporiaceae, and Aspergillaceae) of the phylum Ascomycota. Among these, two strains from the Cladosporiaceae (*Cladosporium* sp. SAAF 22.2.11 and *Cladosporium* sp. 22.3.29) were attractive for the co-occurring parasitoids *T. basalis* and *O. telenomicida*, suggesting a specificity of parasitoid olfactory responses.

Although not much is known about the role played by nectar-inhabiting filamentous fungi on the foraging behavior of flower-associated insects, an increasing body of evidence is accumulating rapidly for yeasts. In olfactometer studies, nectar-fermentation by the specialist nectar yeasts *Metschnikowia* spp. has been shown to elicit attraction in the aphid parasitoid *A. ervi* (Sobhy et al. 2018, 2019) and the egg parasitoids *T. basalis* and *O. telenomicida* (Ermio et al. 2024), which is in agreement with our results on filamentous fungi. Taking together, these results indicate that insect parasitoids display broad olfactory responses to nectar fermented by fungi from the Ascomycota phylum. No negative effects on parasitoid longevity were observed when wasps consumed nectar fermented by *Metschnikowia* spp., suggesting that specialist yeasts do not decrease nectar quality (Sobhy et al. 2018, 2019). To determine whether parasitoid olfactory responses to nectar fermented by filamentous fungi are adaptive, further studies are needed to explore whether feeding on *Cladosporium*-fermented nectar provides a fitness benefit to parasitoids, such as extended longevity or increased fecundity. A possible reason for the absence of olfactory responses by the egg parasitoids towards *Aspergillus* sp.- and *Stachybotrys* sp.-fermented nectars could be explained if the parasitoids experience a fitness cost when consuming such fermented nectar. Some filamentous fungi, such as members of the genus *Aspergillus*, have been shown to be pathogens of hymenopterans (Nicoletti et al. 2024), although little is known about their effects on parasitoids (Dicke et al. 2020). Further studies should also explore whether there is a benefit for the filamentous fungi to attract parasitoids, as it is currently not known whether parasitoids may act as effective dispersal agents of nectar-inhabiting microbes.

In our study, we found that egg parasitoids significantly preferred odors from nectar fermented by two strains from the Cladosporiaceae (*Cladosporium* sp. SAAF 22.2.11 and *Cladosporium* sp. 22.3.29) over odors emitted by the non-fermented control. In our headspace analysis of the nectar fermented by the six filamentous fungi, we detected the following VOCs: 2,6-di-tert-butyl-p-benzoquinone, hydroxyacetophenone, heptadecane, hexadecanoic acid, 2,4-dimethyl glutarate, methyl benzoate and α-terpineol. Among these chemicals, α-terpineol was shown to have electroantennographic activity in the bark beetle parasitoid species *Roptrocerus xylophagorum* (Ratzeburg) (Hymenoptera: Pteromalidae) (Pettersson et al. 2000). Methyl benzoate and 2,6-di-tert-butyl-p-benzoquinone have been reported to have antimicrobial properties(Salvatore et al. 2021) and may thus confer competitive advantages when other microbes colonize the same buckwheat flower. In addition, methyl benzoate is reported to be a plant defensive chemical with eco-friendly botanical-insecticide properties while safe to non-target organisms such as the natural predators coccinellids *Coccinella septempunctata* L. and *Harmonia axyridis* Pallas. Methyl benzoate was reported as the main volatile emitted in the scent of snapdragon flowers(Dudareva et al. 2000), whereas *p*-benzoquinone was found in the headspace of buckwheat flowers (Foti et al. 2017). The compounds acetophenone and hydroxyacetophenone, previously reported to signal nectar sources to parasitoids, were found to enhance plant attractiveness and promote natural pest control (Rohrig et al. 2008). Although it is reasonable to suspect that the above-mentioned compounds could be responsible for the attraction of *T. basalis* and *O. telenomicida* towards nectar fermented by filamentous fungi, this hypothesis should be validated by testing the responses of both parasitoids to standard chemical compounds. Given that attractive and non-attractive nectars emit overall the same compounds, it is possible to hypothesize that the egg parasitoids can detect changes in the ratios of specific volatiles within the blends, adopting the so-called “ratio-specific odor recognition” (McCormick et al. 2012). This strategy has been suggested to play a role in other egg parasitoid species when exploiting herbivore-induced plant volatiles in order to locate their hosts (Cusumano et al. 2015). Furthermore, it would be particularly interesting to investigate the role of hydroxyacetophenone, as this VOC was detected in nectar fermented by *Cladosporium* sp. SAAF 22.2.11 but was absent in *Cladosporium* sp. 22.3.29, suggesting that this compound may be necessary to elicit attraction in *T. basalis*. In contrast, its role may be less relevant for *O. telenomicida*, which was attracted to odors emitted from nectar fermented by both *Cladosporium* strains. Given the widespread occurrence of yeasts in floral nectar, the lack of taxa such as *Metschnikowia* in our study was surprising, as we only detected filamentous fungi. A possible explanation for the absence of nectar-inhabiting yeasts could lie, at least partially, in the community composition of insect pollinators associated with buckwheat: flowers are commonly visited by honeybees but not by bumblebees(Liu et al. 2020), which are considered the main dispersal agents of specialist yeasts such as *Metschnikowia* spp. (Brysch-Herzberg 2004). In fact, nectar-inhabiting yeasts have been shown to increase flower visitation rates, particularly in the case of bumblebees (Herrera et al. 2013; Schaeffer et al. 2014, 2017), whereas honeybees often displayed neutral or even negative foraging responses towards nectar-inhabiting yeasts (Crowley-Gall et al. 2022; Rering et al. 2020). It is also possible to argue that, in the absence of yeasts, floral nectar represents a niche available for colonization by less specialized fungal taxa such as molds in the genera *Cladosporium* and *Aspergillus*. These filamentous fungi can grow in sugar-rich resources and, in some instances, were already reported to occur in floral nectar (Ehlers and Olesen 1997; von Arx et al. 2019) although we currently do not know the actual densities at which filamentous fungi occur in natural nectar. As we are not aware of any other studies that have investigated the microbial composition of buckwheat floral nectar, further research is needed to better understand which microbial taxa dominate and whether the absence of yeasts is common in the nectar of buckwheat flowers.

In this work, we highlight the importance of recognizing that interactions between parasitoids and flowering plants are not simply “bipartite”; instead, we should use a “tripartite” plant-insect-microbe perspective, considering that microbes often serve as hidden players in plant-insect interactions (Colazza et al. 2023; Dicke et al. 2020; Frago et al. 2012). Among nectar-inhabiting microbes, bacteria and yeasts have been increasingly studied in the ecology of pollinators and parasitoids (Cusumano and Lievens 2023; Vannette 2020). By showing that fermentation by filamentous fungi can also affect the foraging behavior of insect parasitoids, we expand the number of microbial taxa known to play a role in the interaction between flower-visiting insects and flowering plants. Finally, our results may also be relevant for sustainable agriculture, as flowering resources such as buckwheat are implemented in agroecosystems to enhance the efficacy of natural enemies as biological control agents of insect pests (Gurr et al. 2017; Heimpel and Mills 2017).

## Electronic Supplementary Material

Below is the link to the electronic supplementary material.

Supplementary Material 1

## Data Availability

All sequences obtained in this study have been deposited in GenBank under the accession numbers PP990345- PP990350. Behavioral and chemical datasets generated and/or analysed during the current study are available from the corresponding author on reasonable request.
