# Supplementary material for: Neglected Microbes in Floral Nectar: Influence of Filamentous Fungi on Nectar Scent and Parasitoid Olfactory Responses
Source: J Chem Ecol. 2025 Mar 12;51(2):33. doi: 10.1007/s10886-025-01586-2 (PMC11903542; doi:10.1007/s10886-025-01586-2)
Supplement: Supplementary file 1 — Supplementary Material 1 [file 10886_2025_1586_MOESM1_ESM.docx]

**SUPPLEMENTARY INFORMATION**

**Fig. S1** Phylogenetic placement of the filamentous fungal strains isolated from buckwheat floral nectar (indicated with orange circles). The phylogenetic tree was inferred from ITS-rDNA sequences from the isolated strains alongside 18 reference sequences obtained from GenBank, using the Neighbour-Joining method (Saitou and Nei 1987). Evolutionary distances were computed using the Jukes-Cantor method (Jukes and Cantor 1969), measured as the number of base substitutions per site. There was a total of 550 positions in the final dataset. Analyses were conducted in MEGA X (Kumar et al. 2018). The percentage of replicate trees in which the associated taxa clustered together in the bootstrap test (1000 replicates) are shown next to the branches


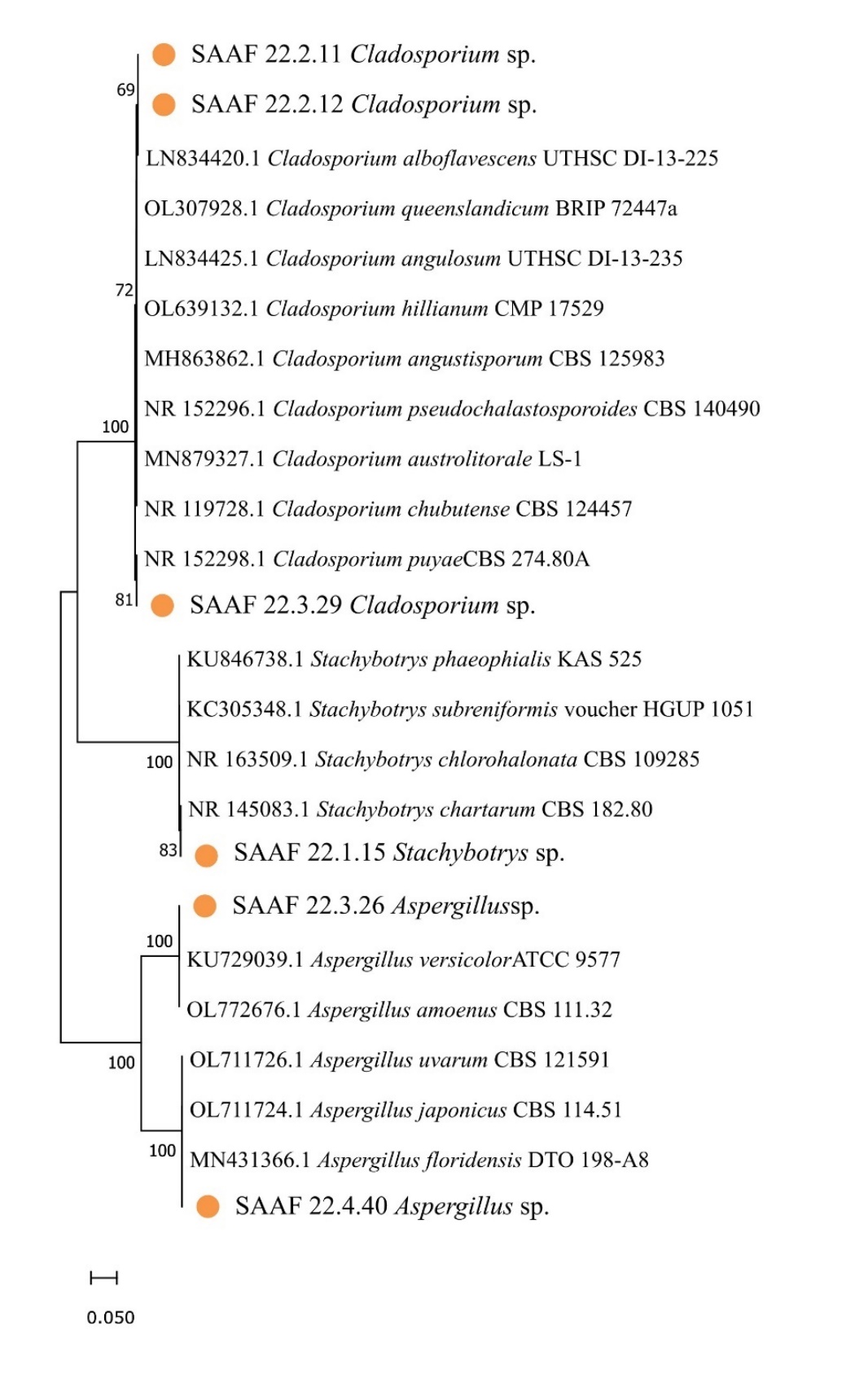


**Table S1** Filamentous fungal strains isolated from buckwheat floral nectar. Identification was based on sequencing the internal transcribed spacer (ITS) region of the ribosomal DNA and comparison with available reference sequences from GenBank with the help of BLASTN. Fungi were assigned to the closest homologous strain according to the highest percentage of sequence identity

| **Strain ID** | **Accession Number**^a^ | **Family** | **Closest match in GenBank** | **Sequence identity (%)** | **Accession no.** |
| --- | --- | --- | --- | --- | --- |
| SAAF 22.2.11 | PP990346 | Cladosporiaceae | *Cladosporium angulosum* UTHSC DI-13 235; | 99.81(532/533) | LN834425.1 |
| SAAF 22.2.12 | PP990347 | Cladosporiaceae | *Cladosporium queenslandicum* BRIP 72447a | 99.81 (525/526) | OL307928.1 |
| SAAF 22.3.29 | PP990348 | Cladosporiaceae | *Cladosporium puyae* CBS 274.80A | 100 (519/519) | NR152298.1 |
| SAAF 22.3.26 | PP990349 | Aspergillaceae | *Aspergillus versicolor* ATCC 9577 | 100 (505/505) | OL772676.1; KU729039.1 |
| SAAF 22.4.40 | PP990350 | Aspergillaceae | *Aspergillus uvarum* CBS 121591 ; *Aspergillus japonicus* CBS 114.5; 1 *Aspergillus floridensis* DTO_198-A8 | 100 (578/578) | OL711726.1; OL711724.1; MN431366.1 |
| SAAF 22.2.15 | PP990345 | Stachybotryaceae | *Stachybotrys phaeophialis* KAS 525 *Stachybotrys subreniformis* voucher HGUP 1051 | 99 (575/577); | KU846738.1;  KC305348.1 |

*^a^GenBank accession numbers of the partial sequences of the ITS ribosomal DNA of the fungal strains isolated in this study*
